# Blue Rubber Bleb Nevus Syndrome Complicated by Enhanced-Fibrinolytic-Type DIC: A Case Report

**DOI:** 10.3400/avd.cr.20-00148

**Published:** 2021-09-25

**Authors:** Shinya Yamada, Masahisa Arahata, Eriko Morishita, Hidesaku Asakura

**Affiliations:** 1 Department of Hematology, Kanazawa University Hospital, Kanazawa, Ishikawa, Japan

**Keywords:** blue rubber bleb nevus syndrome, disseminated intravascular coagulation, fibrinolytic activation

## Abstract

A 54-year-old Japanese man was diagnosed with blue rubber bleb nevus syndrome (BRBNS) due to venodilation in the lower extremities at birth and gastrointestinal vascular malformations. He also had small bowel bleeding and enhanced-fibrinolytic-type disseminated intravascular coagulation (DIC). Endoscopic sclerotherapy for intestinal hemangioma could not be performed because of bleeding concerns; instead, a combined anticoagulant and antifibrinolytic treatment was performed. Although combination treatment with unfractionated heparin and tranexamic acid proved ineffective for small bowel bleeding, combination treatment with apixaban and tranexamic acid dramatically improved enhanced-fibrinolytic-type DIC. In BRBNS, treatment strategies should be considered after performing detailed coagulation tests.

## Introduction

Blue rubber bleb nevus syndrome (BRBNS) is a venous malformation first reported by Gascoyen in 1860^1)^ and named by Bean in 1958.^2)^ The presence of hemangiomas in the skin with aging is one of the predominant symptoms. BRBNS may cause hemangiomas in the central nervous system, liver, spleen, kidney, lung, heart, thyroid, or muscle. In addition, because of multiple vascular malformations in the gastrointestinal tract, various degrees of gastrointestinal bleeding and iron-deficiency anemia may be observed. Approximately 200 cases of BRBNS have been reported worldwide.^3)^ However, reports of the association between BRBNS and coagulation abnormalities or disseminated intravascular coagulation (DIC) are extremely rare.^4)^ We report a case of BRBNS with persistent severe gastrointestinal bleeding complicated by enhanced-fibrinolytic-type DIC^5)^ in which combination treatment involving a direct oral anticoagulant (DOAC) and tranexamic acid proved effective.

## Case Report

A 54-year-old Japanese man presented with a history of varices in the lower extremities noticed at birth. At 5 years old, he had been diagnosed with congenital phlebectasia and had been followed up without intervention. Arthrocentesis performed at 25 years old for fever and left knee pain had caused massive bleeding within the knee joint. A checkup at 45 years old revealed anemia (hemoglobin (Hb), 10.3 g/dL) and he visited our hospital for detailed examination. Abnormalities were identified in the coagulative and fibrinolytic pathways, so only an iron preparation was prescribed for anemia, and he was followed up.

**Figure 1:**
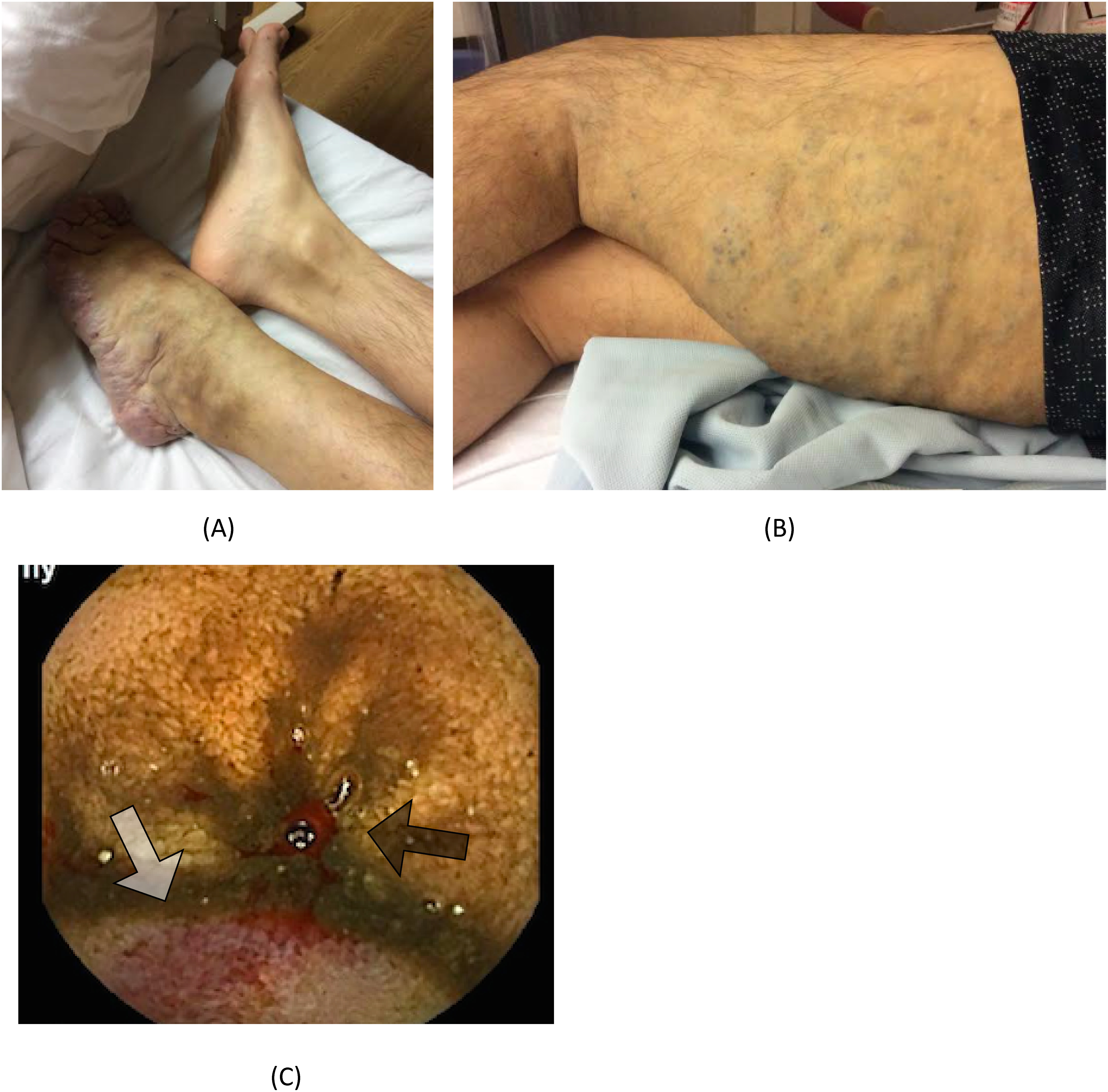
Fig. 1 Elastic, black-toned angiomatous lesions of 0.5–2 cm in areas (**A**) surrounding the joint in the left foot and (**B**) from the left buttock to the dorsal thigh. (**C**) Capsule endoscopy shows oozing bleeding (black arrow) and dilated vein (white arrow) at ileum. Informed consent was obtained from the patient for publication of the photographic materials.

On this presentation, unconsciousness, fever, and advanced anemia were observed, and the patient was urgently hospitalized. Examination showed white blood cell count, 8,870/µL; Hb, 9.0 g/dL; platelet count, 172,000/µL; prothrombin time (PT), 12.6 s (reference, 10.1–12.7 s); activated partial thromboplastin time (APTT), 28.3 s (reference, 24.0–37.7 s); fibrinogen (Fbg), 100 mg/dL (reference, 200–400 mg/dL); fibrin/fibrinogen degradation products (FDP), 427.9 µg/mL (reference, <5.0 µg/mL); D-dimer, 183.8 µg/mL (reference, <1.0 µg/mL); antithrombin (AT), 89% (reference, 70%–130%); thrombin–antithrombin complex (TAT), 31.5 ng/mL (reference, <3.9 ng/mL); plasmin–α_2_ plasmin inhibitor (α_2_PI) complex (PIC), 8.0 µg/mL (reference, <0.8 µg/mL); plasminogen, 72% (reference, 70%–130%); and α_2_PI, 39% (reference, 70%–130%). A blood culture detected *Escherichia coli*, and magnetic resonance imaging (MRI) showed discitis of the L1/L2 intervertebral disc. During physical examination, conjunctival pallor and knocking pain at the midpoint of the lower back were observed. Dilated veins were evident from the left buttock to the left lower extremity (**Fig. 1**). No subcutaneous bleeding or other forms of bleeding were observed.

**Figure 2:**
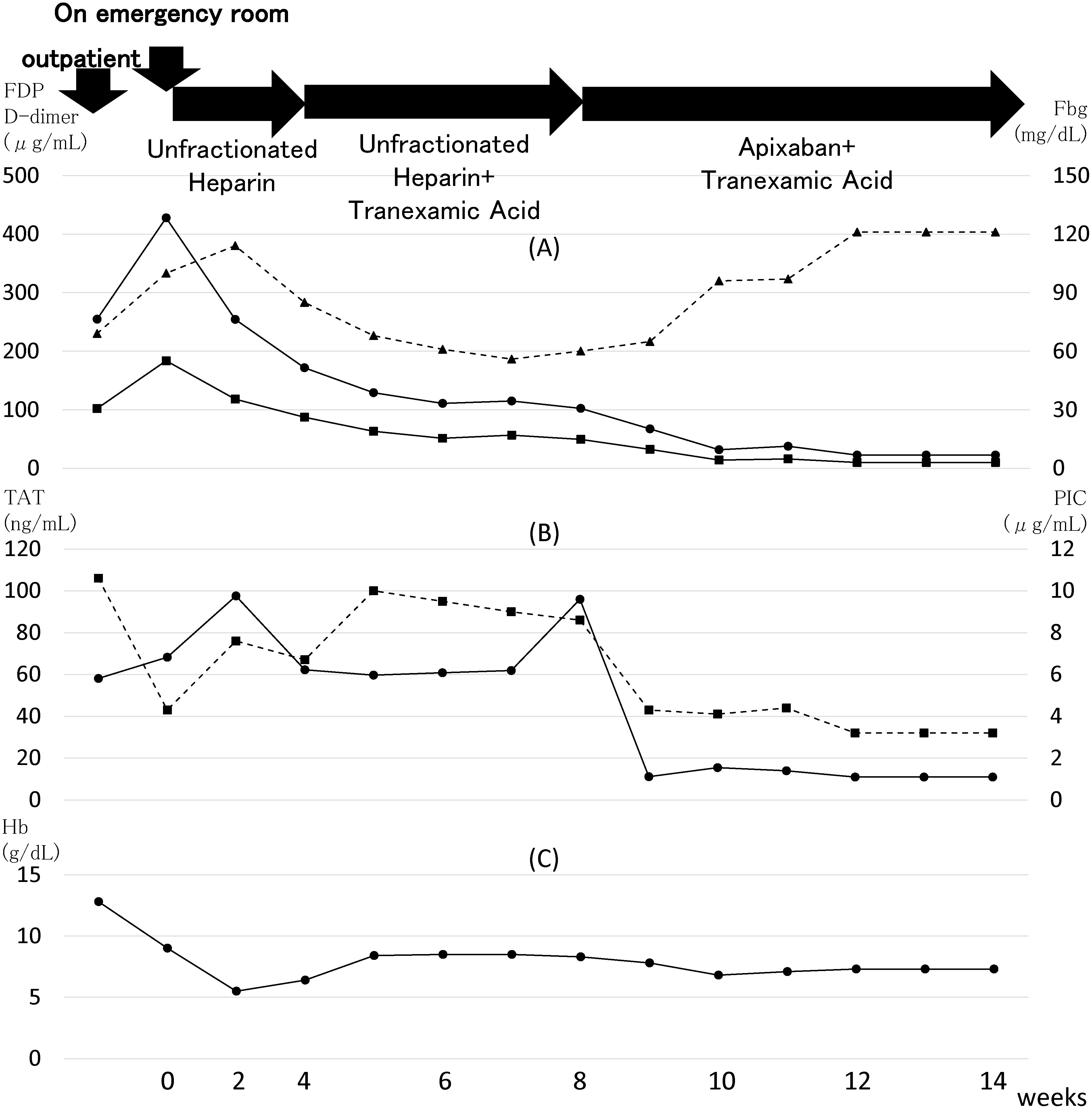
Fig. 2 Changes in results of coagulation testing and hemoglobin (Hb). Numbers at the bottom represent the length of hospital stay in weeks, with time on admission set as 0. (**A**) Changes in fibrin/fibrinogen degradation products (FDP), D-dimer, and fibrinogen (Fbg). ●: FDP; ■: D-dimer; ▲: Fbg. FDP and D-dimer temporarily decreased after starting treatment with unfractionated heparin alone, and both decreased further after switching to apixaban. Fbg temporarily increased because of the presence of infection at the time of admission to our hospital but decreased as infection improved. During the period of combination treatment with unfractionated heparin and tranexamic acid, Fbg was kept at approximately 60 mg/dL but clearly increased after starting combination treatment with apixaban and tranexamic acid. (**B**) Changes in thrombin–antithrombin complex (TAT), a marker for the activation of coagulation, and plasmin–α_2_ plasmin inhibitor (α_2_PI) complex (PIC), a marker for the activation of fibrinolysis. ●: TAT; ■: PIC. Neither TAT nor PIC decreased clearly until combination treatment with apixaban and tranexamic acid was started. (**C**) Changes in Hb. Despite the changes shown in (**A**) and (**B**), the improvement in anemia is poor and red blood cell transfusions (280 mL each) have been required once every two or three days.

At this point, the patient was considered to have DIC as a complication of *E. coli* sepsis and discitis in addition to primary enhanced-fibrinolytic-type DIC caused by varices. We thus initiated treatment with continuous infusion of cefazolin, sulfamethoxazole trimethoprim mixture, and unfractionated heparin (peripheral intravenous drip infusion at 500 units/kg/24 h). The clinical course is shown in **Fig. 2**. Unconsciousness improved the day after admission, and C-reactive protein declined from 2.3 to <1.0 mg/dL over the course of 1 week, suggesting that infection was controlled. Though platelet count remained unchanged at approximately 150,000/µL, PIC increased to 11.2 µg/mL and Hb decreased to 5.5 g/dL on day 7 of admission. Capsule endoscopy revealed oozing bleeding from the small intestine and vascular malformations in the stomach, small intestine, and colon (**Fig. 1C**). Bleeding was thought to be caused by both BRBNS and enhanced-fibrinolytic-type DIC. Vascular lesions were evident in the skin and gastrointestinal tract, measuring 0.1–5 cm in diameter, blue to black in color, and with a rubbery texture. We therefore diagnosed the vascular malformations as BRBNS. Although we considered sclerotherapy with double-balloon endoscopy as a potential treatment for small bowel bleeding, the background presence of severe enhanced-fibrinolytic-type DIC resulted in the risk of bleeding being considered too high; therefore, this treatment was not implemented. We also considered endovascular treatment. However, identifying the vessel responsible for bleeding was considered difficult and vascular embolization of a wide area would risk small bowel necrosis, so this treatment was likewise not conducted. We therefore decided to address the small bowel bleeding via treatment of DIC. First, we performed treatment with unfractionated heparin (500 units/kg/24 h) alone. However, significant increases in both TAT and PIC continued and severe melena persisted, so the treatment was judged ineffective. Next, we performed combination treatment with heparin (500 units/kg/24 h) and tranexamic acid (500 mg per os (p.o.) quater in die (QID)), as a treatment that has been reported as highly effective for enhanced-fibrinolytic-type DIC.^6)^ DIC examination results still did not improve (fibrinogen levels remained at approximately 60 mg/dL), and severe melena also persisted. In addition, ultrasound of the lower extremity veins showed deep venous thrombosis at soleus veins on day 50. Unfractionated heparin was thus switched to a DOAC, apixaban, implementing combination treatment with apixaban (5 mg p.o. bis in die (BID)) and tranexamic acid (500 mg p.o. QID). Switching from combination treatment with unfractionated heparin and tranexamic acid to combination treatment with apixaban and tranexamic acid dramatically improved DIC examination results (**Figs. 2A** and **2B**). In particular, sharp drops in TAT and PIC levels due to the switch in treatments and clear recovery of fibrinogen levels were impressive. However, melena persisted despite the dramatic improvement in DIC examination results, and regular red blood cell transfusions are still required as of the time of writing. The patient is currently in a condition where another strategy for hemostasis is required (**Fig. 2C**) and still needs hospitalization.

## Discussion

The present case involved a patient with BRBNS who experienced DIC as a complication. Although approximately 200 cases of BRBNS have been reported, results of coagulation tests were described for few of those patients. The incidence of DIC in patients with BRBNS thus remains unknown. In the present patient, elevated TAT (a marker for activation of coagulation), elevated PIC (a marker for activation of fibrinolysis), significantly elevated FDP, elevated the FDP/D-dimer ratio, significantly reduced fibrinogen, and significantly reduced α_2_PI were observed. BRBNS was thus considered to have been complicated by typical enhanced-fibrinolytic-type DIC.^5)^

Kasabach–Merritt syndrome, which is an angiomatosis like BRBNS, is often complicated by DIC.^7)^ On the basis of the mechanism by which Kasabach–Merritt syndrome causes coagulopathy, platelets are considered to be trapped by angiomatous lesions, leading to the activation of coagulation and localized intravascular coagulation, resulting in an exhaustive reduction in coagulation factors.^8)^ Although no studies have reported the mechanisms by which BRBNS causes coagulopathy, similar to Kasabach–Merritt syndrome, blood stasis within hemangiomas may cause consumption of coagulation factors that results in coagulopathy via DIC.

The cornerstone of DIC management involves treatment of the underlying disease.^9)^ However, BRBNS presents with multiple lesions, in which radical treatment via surgical resection is difficult and topical treatment via endoscope such as sclerotherapy, electrocautery, and mucosectomy is commonly performed. In enhanced-fibrinolytic-type DIC, lesions are extremely hemorrhagic, and treatment can cause serious, life-threatening massive bleeding. In BRBNS such as the present case, vessels are extremely fragile in addition to the DIC, and induction of massive bleeding via topical treatment is a concern. We therefore decided to stop bleeding using anticoagulation and antifibrinolytic treatment for DIC. Although unfractionated heparin alone was used for treatment in the beginning, we started concomitant tranexamic acid on day 27 due to persistent severe melena. However, even after combination with tranexamic acid, no improvement at all was observed in coagulation test results (FDP, ≥100 µg/mL; D-dimer, ≥50 µg/mL; Fbg, ≤60 mg/dL; TAT, ≥60 ng/mL; PIC, approximately 10 µg/mL). Melena also did not improve. Because ultrasound of the lower extremity veins showed deep vein thrombosis (DVT) on day 50, the anticoagulant was switched from unfractionated heparin to the DOAC apixaban, leading to reductions in FDP (approximately 20 µg/mL), D-dimer (approximately 10 µg/mL), TAT (approximately 10 ng/mL), and PIC (approximately 3.0 µg/mL) and an elevation in Fbg (≥120 mg/dL). Because clear reductions were observed, particularly in TAT as a marker for the activation of coagulation, and PIC, a marker for activation of fibrinolysis, DIC was considered to have improved in terms of coagulation. Heparin generally inhibits free activating factor X dependent on antithrombin but is incapable of inhibiting factor Xa in the prothrombinase complex. In contrast, the DOAC apixaban is capable of inhibiting not only free activating factor X but also factor Xa in the prothrombinase complex,^10)^ and this may have resulted in the difference in the effectiveness against DIC. Even after clear improvements in test results for DIC, anemia and gastrointestinal bleeding were prolonged and the patient has remained in a condition where another strategy is required.

In conclusion, to the best of our knowledge, this is the first case report globally to clearly demonstrate BRBNS complicated by enhanced-fibrinolytic-type DIC. In addition, although neither heparin nor combination treatment with heparin and tranexamic acid proved effective for enhanced-fibrinolytic-type DIC, the fact that switching to combination treatment with the DOAC apixaban and tranexamic acid dramatically improved the effectiveness for DIC in terms of coagulation should be noted.
